# RNase L Is Involved in Liposaccharide-Induced Lung Inflammation

**DOI:** 10.3390/v12010073

**Published:** 2020-01-07

**Authors:** Ruhan Wei, Guanmin Chen, Naseh Algehainy, Chun Zeng, Chunfang Liu, Hongli Liu, Wendy Liu, Dennis Stacey, Aimin Zhou

**Affiliations:** 1 Clinical Chemistry Program, Department of Chemistry, Cleveland State University, Cleveland, OH 44115, USA; r.wei17@vikes.csuohio.edu (R.W.); g.chen3@vikes.csuohio.edu (G.C.); n.algehainy@vikes.csuohio.edu (N.A.); davina_zeng@hotmail.com (C.Z.); Liuhonglili@hotmail.com (H.L.); 2 Institute of Chinese Materia Medica, China Academy of Chinese Medical Sciences, Beijing 100700, China; chunfang666@126.com; 3 Department of Pathology, University Hospitals Cleveland Medical Center, Cleveland, OH 44115, USA; Wendy.Liu@UHhospitals.org; 4 Department of BGES, Cleveland State University, Cleveland, OH 44115, USA; d.stacey@csuohio.edu; 5 Center for Gene Regulation in Health and Diseases, Cleveland State University, Cleveland, OH 44115, USA

**Keywords:** RNase L, LPS, Acute lung injury, TLR4

## Abstract

RNase L mediates interferon (IFN) function during viral infection and cell proliferation. Furthermore, the role of RNase L in the regulation of gene expression, cell apoptosis, autophagy, and innate immunity has been well established in the last decade. Tissue distribution reveals that RNase L is highly expressed in the lung and other organs. However, the physiological roles of RNase L in the lung are largely unknown. In this study, we found that polysaccharide (LPS)-induced acute lung injury (ALI) was remarkably intensified in mice deficient in RNase L compared to wild type mice under the same condition. Furthermore, we found that RNase L mediated the TLR4 signaling pathway, and regulated the expression of various pro- and anti-inflammatory genes in the lung tissue and blood. Most importantly, RNase L function in macrophages during LPS stimulation may be independent of the 2-5A system. These findings demonstrate a novel role of RNase L in the immune response via an atypical molecular mechanism.

## 1. Introduction

Both acute lung injury (ALI) and acute respiratory distress syndrome (ARDS) are characterized by an excessive inflammatory response within the lungs under endotoxin stimulation, leading to severely impaired gas exchange, endothelial-alveolar epithelium injury, alveolar-capillary barrier disruption and pulmonary edema; with high mortality rates from 25% to 45%. ALI/ARDS affects one in 10 general intensive care unit patients; and there are about 200,000 cases annually in the United States [1,2,3]. It is believed that macrophages may be one of the most important immune cells involved in initiating the inflammatory response by releasing proinflammatory cytokines to promote inflammation in the lung [4,5]. To date, there are no effective medicines available for the treatment of ALI/ARDS, which highlights an urgent need to develop novel therapeutic strategies for this disorder.

RNase L mediates the 2-5A system in interferon (IFN) functions against viral infection and cellular proliferation [6]. The two major enzymes involved in the 2-5A system are 2′-5′-oligoadenylate synthetase (OAS) and RNase L. Type I IFNs initiate transcription of the OAS genes to produce a family of OAS isoforms. OAS proteins can be activated by double-stranded RNA (dsRNA) that is frequently a viral pathogen-associated molecular pattern. After activation by dsRNA, OAS converts ATP molecules to pyrophosphate (ppi) and a series of unique, 5′-phosphorylated, 2′-5′ linked oligoadenylates known as 2-5A, with the general formula ppp(A2′p5′)nA (*n* ≥ 2). 2-5A binds human RNase L with high affinity, converting it from its inactive, monomeric state to a potent dimeric endoribonuclease, resulting in degradation of single-stranded (ss) viral and cellular RNAs [7]. Accordingly, it has been demonstrated that 2-5A accumulates and RNase L is activated in intact infected cells. Cells overexpressing RNase L were better able to overcome viral infection [8]. In contrast, overexpression of a dominant negative mutant of RNase L results in increased susceptibility to certain viruses [9]. In vivo studies show that mice containing targeted disruption of the RNase L gene succumb to encephalomyocarditis (EMCV) infection more rapidly than wild type mice [10]. Furthermore, studies reveal that RNase L also plays an important role in the regulation of gene expression at both transcriptional and translational levels, cell apoptosis, and virus-induced autophagy through different mechanisms depending the specific RNA substrates and the extent of ribonuclease activity [11,12,13,14]. It has been demonstrated that active RNase L cleaves ssRNAs, both cellular and viral, in a variety of cell types. This can result in the generation of small RNAs capable of activating retinoic acid-inducible gene-I (RIG-I)-like receptors, or the nucleotide-binding oligomerization domain-like receptor 3 (NLRP3) inflammasome, leading to innate immune responses [15,16].

Accumulating evidence shows that RNase L may have functions outside of its known anti-viral roles. Tissue distribution analysis has revealed that RNase L is highly expressed in the spleen, thymus, and most of the immune cells such as T, B cells and macrophages, suggesting a role for RNase L in the immune system [17]. Indeed, RNase L null mice show enlarged thymus glands and increased T cell numbers at an early age, indicating that RNase L may be involved in T cell development [10]. Suppressed skin allograft rejection [18] and severely impaired alphavirus-based DNA vaccination against a non-mutated tumor-associated self-antigen (tyrosinase-related protein-1, TRP-1) were observed in RNase L deficient mice, indicating that RNase L plays an important role in the host immune system [19]. In our previous study, we have demonstrated that RNase L is involved in macrophage function using bone marrow-derived macrophages (BMMs) from RNase L^+/+^and ^−/−^ mice. Deficiency of RNase L remarkably decreased the migration of BMMs induced by M-CSF, but to a reduced extent by GM-CSF and chemokine C-C motif ligand-2 (CCL2). Interestingly, RNase L was found to mediate endocytic activity of macrophages and regulate the expression of pro-and anti-inflammatory genes, such as TGF-β, IL-1β, IL-10, CCL2, and Cox-2, induced by different stimuli [20]. Apparently, the involvement of RNase L in these cell functions may or may not be through its endonuclease activity.

In this study, we found that mice deficient RNase L showed intensified ALI induced by LPS. Further investigation of the molecular mechanism revealed that RNase L regulated the expression of pro- and anti-inflammatory genes through mediating the TLR4 signaling pathway. This suggests that RNase L function in LPS-induced immune responses may be independent of its nuclease activity. Our results have shown a novel role of RNase L in microbial immunity.

## 2. Materials and Methods

### 2.1. Reagents and Antibodies

Antibodies against p-ERK, ERK1/2, ERK, p-IκB-α, p-c-Jun, p-JNK, and TLR4 were from Santa Cruz Biotechnology, Inc. (Dallas, TX, USA). The antibodies for Cox-2 and p-IRF3 were from Cayman Chemical (Ann Arbor, MI, USA) and Cell Signaling (Danvers, MA, USA). ELISA kits for TNF-α, IL-1β. IL-6, IL-4 and IL-10 were from Thermo Fisher (Carlsbad, CA, USA). Murine RNase L polyclonal antibody was produced by our lab.

### 2.2. Knocking Down of RNase L in Raw 264.7 Cells

Raw 264.7 cells, a murine macrophage cell line, were cultured in a 12-well plate to 50% confluency on the day of infection. Complete medium with polybrene from Santa Cruz (Dallas, TX, USA) at a final concentration of 5 µg/mL was added to each well and cells then were infected by directly adding mouse RNase L shRNA or empty lentiviral particles from Santa Cruz (Dallas, TX, USA). After incubation overnight, the culture medium was replaced with a fresh complete medium. Clones were selected by culturing the infected cells in the medium containing puromycin (10 µg/mL) and expression of RNase L in an individual clone was analyzed by Western blot.

### 2.3. Animal Treatment

Age-matched male RNase L wild type and knockout mice (*n* = 6) (a generous gift from Dr. Robert Silverman, Cleveland Clinic) were anesthetized by intramuscular injection of ketamine/xylazine (50 mg/kg) admixture, followed by intranasal instillation of LPS (40 μg in 50 μL PBS). Mice with only 50 µL PBS were used as control. After LPS inhalation for 24 h, mice were sacrificed, and the lung tissues and cornea blood were harvested. This study was carried out in strict accordance with the recommendations in the Guide for the Care and Use of Laboratory Animals of the National Institutes of Health. The protocol was approved by the Committee on the Ethics of Animal Experiments of Cleveland State University (Permit Number: 21149-ZHO-AS). All efforts were made to reduce suffering. Mice were under CO_2_ for 5 min and then dislocated their neck at the termination of the experiments.

### 2.4. Analysis of RNase L-Mediated rRNA Cleavages in Intact Cells

The RNase L nuclease activity was analyzed using RNA chips. RNase L wild type and knockdown Raw 264.7 cells were infected with vaccinia virus at different multiplicity of infection (MOI) or 1 µg/mL LPS for 16 h. Total RNA was isolated from the cells using the Trizol reagent (Invitrogen, Grand Island, NY, USA) according to manufacturer’s instruction. RNA (1 µg) was separated on RNA chips and analyzed with an Agilent Bioanalyzer 2100 (Agilent, Palo Alto, CA, USA). The peak areas of 28S and 18S rRNA and their main cleavage products were measured using the Bio Sizing program Version A.02.01 S1232 (Agilent, Palo Alto, CA, USA).

### 2.5. Western Blot Analysis

After treatment, cells were washed twice with ice-cold PBS and collected with a scraper. The cytoplasmic extracts were prepared by suspension of the cell pellets in the NP-40 lysis buffer (10 mM Tris-HCl, pH 8.0, 5 mM Mg(OAc)_2_, 90 mM KCl, 0.2 mM PMSF, 100 units/mL aprotinin, 10 µg/mL leupeptin and 2% NP-40). After centrifugation at 10,000× *g* in a microcentrifuge at 4 °C for 10 min, the cell extracts (100 µg per sample) were fractionated on 10% SDS-polyacrylamide gels and transferred to PVDF membranes (Millipore, Billerica, MA, USA). The membranes were blocked with 5% nonfat milk in PBS containing 0.02% sodium azide and 0.2% (*v*/*v*) Tween 20, and incubated with different primary antibodies for 1 h at room temperature. The membranes were then washed with PBS containing 0.2% (*v*/*v*) Tween 20 and incubated with specific secondary antibodies conjugated with horseradish peroxidase (Cell Signaling, Billerica, MA, USA) for 1 h at room temperature. After washing, the proteins were detected by a chemiluminescent method according to the manufacturer’s specification (Pierce, Rockford, IL, USA).

### 2.6. Histostaining

The lungs dissected from the mice were fixed in 10% formalin, paraffin embedded, and sectioned at 5 µm, prior to staining with hematoxylin and eosin. The images were taken with an Olympus BX40 microscope (Center Valley, PA, USA) at Akron Children Hospital (Akron, OH, USA).

### 2.7. Enzyme-Linked Immunosorbent Assay (ELISA)

Cytokines and chemokines in the cornea blood and lung tissue extracts were measured by ELISA with commercially available kits (Thermo Fisher, Carlsbad, CA, USA). Briefly, flat bottom 96-well ELISA plates were coated with a capture antibody at 4 °C according to manufacturer’s instruction. After overnight incubation at 4 °C, the plates were washed three times and blocked with the blocking buffer provided in the kit, and then incubated with standards and samples for 2 h at room temperature. After washing the plates, a specific biotinylated antibody was added to each well and incubated for 1 h, followed by washing and 30 min incubation with avidin peroxidase. Then, substrates containing 3, 3′ 5, 5′-tetramethylbenzidine (TMB) and hydrogen peroxide were added, and the reaction was terminated by adding 50 μL of phosphoric acid after 30 min. Plates were read at 450 nm in a 96-well LD 400C microplate reader (Beckman Coulter, Fullerton, CA, USA).

## 3. Results

### 3.1. Deficiency of RNase L Increases Lung Tissue Damage in LPS-Induced ALI

**Figure 1:**
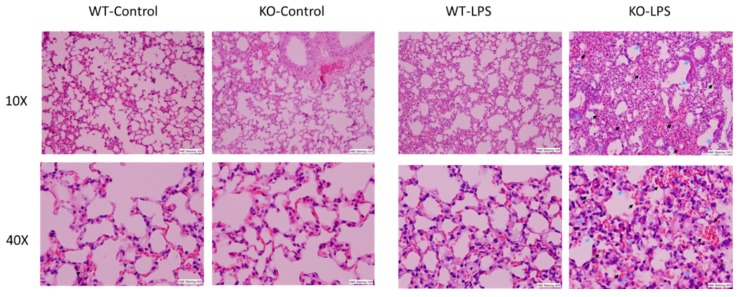
LPS-induced more severe lung pathological alterations in RNase L deficient mice compared to wild type mice. Age-matched male RNase L wild type and deficient mice (6/group) received intranasal instillation of LPS for 24 h. Histological changes in the lung structures after LPS inhalation are shown. Representative images of hematoxylin and eosin stained sections of lung tissues are present at 10× and 40× magnifications. Pneumocytes (indicated by blue arrows) and hemorrhage (indicated by black arrows).

Alveolar macrophages (AM) play a critical role in the development of ALI through the synthesis and release of a variety of inflammatory mediators in the lung upon infection or non-infectious stimulation. Our previous results have demonstrated that RNase L contributes to macrophage function. To determine the involvement of RNase L in LPS-induced lung injury, the lung tissues of mice were stained with hematoxylin and eosin for histological assessment, after receiving an intranasal instillation of LPS for 24 h. As shown in Figure 1, LPS treatment induced pathological alterations, including hemorrhage, collapsed alveolar spaces, interstitial and intra-alveolar edema, pneumocyte hyperplasia and inter-alveolar septal thickening in both RNase L knockout and wild type mice, but no significant immune cell infiltration was observed. However, the morphological abnormalities seen in the lung of RNase L knockout mice were much more severe, suggesting that RNase L may play an important role in protecting the lung from LPS-induced damage.

### 3.2. RNase L Regulates the Expression of Pro-and Anti-Inflammatory Genes in the Lung and Blood upon LPS Stimulation

**Figure 2:**
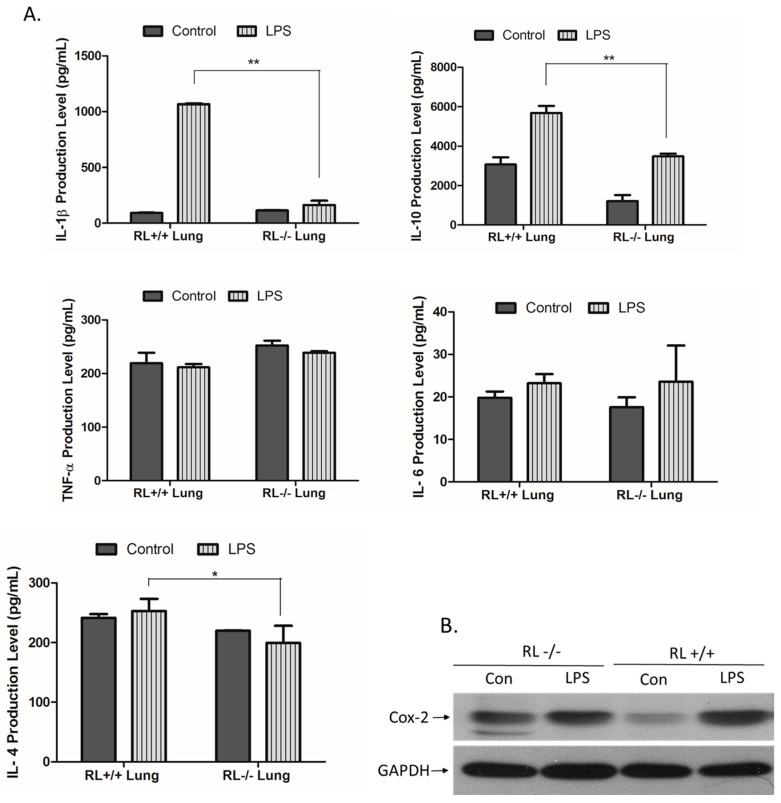
Expression of pro- and anti-inflammatory genes in the lung tissues. (**A**) Lung tissue extracts obtained from RNase L wild type and deficient mice received intranasal instillation of LPS for 24 h. The level of certain cytokines and chemokines in the lung tissue extracts was measured with an ELISA kit for each of the markers. Experiments were performed twice in triplicates. Data are presented as mean ± SD. * *p* < 0.045, ** *p* < 0.004. (**B**) The level of Cox-2 in the lung extracts was determined by Western blot with an antibody against mouse Cox-2 (Cayman, MI, USA).

**Figure 3:**
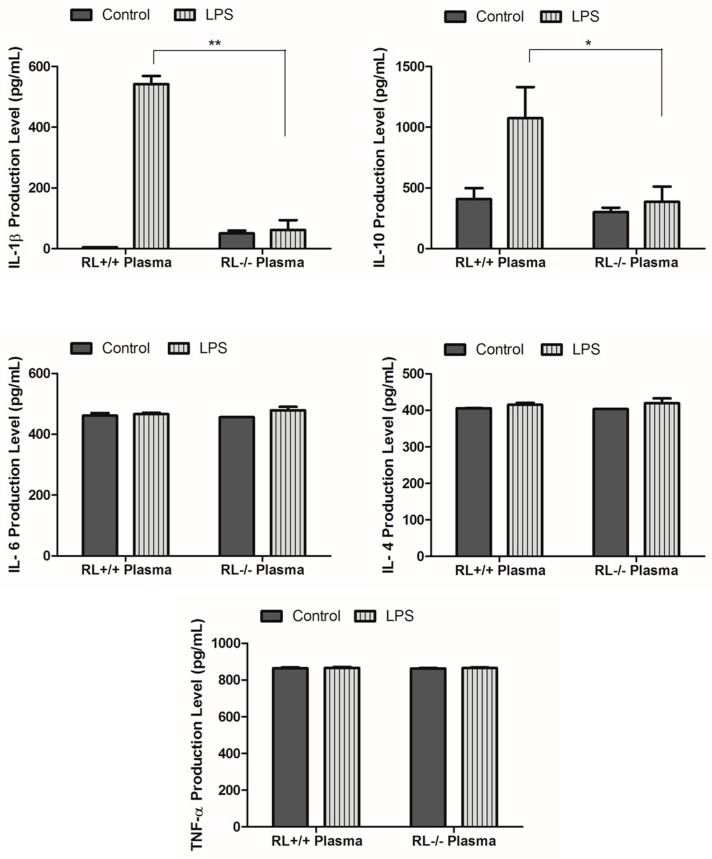
Levels of certain cytokines and chemokines in the plasma. Cornea blood was isolated from RNase L wild type and deficient mice that received intranasal instillation of LPS for 24 h. The level of certain cytokines and chemokines in the plasma was measured using an ELISA kit for each of the indicated markers. Experiments were performed twice in triplicates. Data are presented as mean ± SD. * *p* < 0.05, ** *p* < 0.001.

RNase L regulates the expression of pro- and anti-inflammatory genes in macrophages induced by various stimuli. To determine the effect of RNase L on the development of ALI, the differential expression of certain pro- and anti-inflammatory genes such as TNF-α, IL-1β, IL-4, IL-6, IL-10, and Cox-2 in the lung tissues and blood from RNase L wild type and knock out mice after LPS administration was examined by ELISA and Western blot analyses. In lung tissues, the levels of IL-10 and IL-1β were remarkably increased. The level of IL-10 was significantly higher in the lungs of RNase L wild type mice compared to RNase L deficient mice after treated with LPS (Figure 2A). In addition, the level of IL-6 was slightly increased in the lung tissues of both mouse types after treatment with LPS. Western blot analyses revealed that the expression of Cox-2 was significantly higher in the lung tissues of RNase L wild type mice induced by LPS, although the Cox-2 basal level in the lungs from RNase L deficient mice was higher (Figure 2B). Interestingly, the levels of IL-4, IL-6, and TNF-α were not significantly changed in the blood of RNase L wild type mice compared to RNase L deficient mice after treated with LPS; a similar observation was found in the lung tissues. On the other hand, the levels of IL-10 and IL-1β were dramatically increased in RNase L wild type mice (Figure 3).

### 3.3. RNase L Mediates the TLR4 Signaling Pathway Activated by LPS in Macrophages

**Figure 4:**
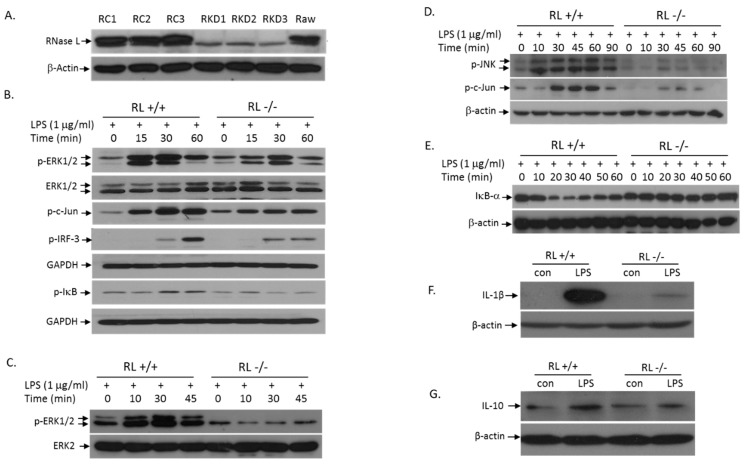
Absence of RNase L attenuates LPS-activation of the TLR4 signaling pathway. RNase L was knocked down in Raw 264.7 cells with RNase L shRNA as described in Methods. Empty lentiviral particles (Santa Cruz, Dallas, TX, USA) served as control. The expression of RNase L in control (Raw control: RC1-3) and knockdown (Raw knockdown: RKD1-3) clones was determined by Western blot with a polyclonal antibody against mouse RNase L (**A**). RNase L wild type and knockdown Raw 264.7 cells were treated with 1 µg/mL of LPS for various times, and activation of the downstream targets in the TLR4 signaling pathway such as p-ERK, p-c-Jun, p-IκB and p-IRF-3 was analyzed (**B**). Primary RNase L^+/+^ and ^−/−^ MEFs with the C57BL/6 background were treated with 1 µg/mL of LPS for various times. Activation of p-ERK (**C**), p-JNK and p-c-Jun (**D**), and IκB (**E**) was determined by Western blot. Antibodies used in the experiments were p-JNK (Cell Signaling, Danvers, MA, USA), p-Jun, p-ERK, ERK1/2, ERK2, GAPDH and IκB (Santa Cruz, Dallas, TX, USA), and β-actin (Cayman, Ann Arbor, MI, USA). RNase L wild type and knockdown Raw 264.7 cells were treated with 1 μg/mL of LPS for 14 h. The levels of IL-1β (**F**) and IL-10 (**G**) in cell extracts were analyzed by Western blots with antibodies against mouse IL-1β and IL-10 (Santa Cruz, Dallas, TX, USA). Each of the experiments was performed at least three times.

LPS treatment of cells activates the TLR4 signaling pathway, which induces the expression of pro- and anti-inflammatory cytokines, leading to initiation and development of inflammation. TLR4 transduces signals through the MyD88-dependent and -independent pathways. To determine the role of RNase L in LPS-activation of the MyD88-dependent signaling pathways, we performed Western blot analyses to assess the levels of p-ERK, p-JNK, and p-IκB in Raw 264.7 cells with or without RNase L. First, we used RNase L shRNA in lentiviral particles to knock down RNase L in Raw 264.7 cells, a murine macrophage cell line. The RNase L expression in several selected clones was almost completely suppressed (Figure 4A). We next determined the effect of RNase L on LPS-induced activation of the TLR4 signaling pathway. As shown in Figure 4B, deficiency of RNase L significantly reduced the LPS-activation of the MAPK signaling pathways including ERK and JNK. Consequently, activation of c-Jun, one of the downstream targets of the MAPK pathways, was also reduced in RNase L knockdown Raw 264.7 cells. Furthermore, the activation of the IκB/NFκB pathway by LPS was downregulated in cells lacking RNase L as well. It has been well established that the MyD88-independent pathway can be activated in macrophages by LPS. Obviously, as shown in Figure 4B, RNase L is involved in the MyD88-independent pathway, because the activation of IRF3 was reduced in RNase L knockdown cells following LPS stimulation. We have also obtained the similar results in primary mouse embryonic fibroblasts (MEFs) under the same condition (Figure 4C–E). In addition, deficiency of RNase L significantly reduced the expression of IL-1β and IL-10 in Raw 264.7 cells following LPS stimulation (Figure 4F,G), further demonstrating the role of RNase L in the TLR4-mediated immune responses.

### 3.4. RNase L Function in LPS-Induced Lung Injury May Be Independent of Its Nuclease Activity

**Figure 5:**
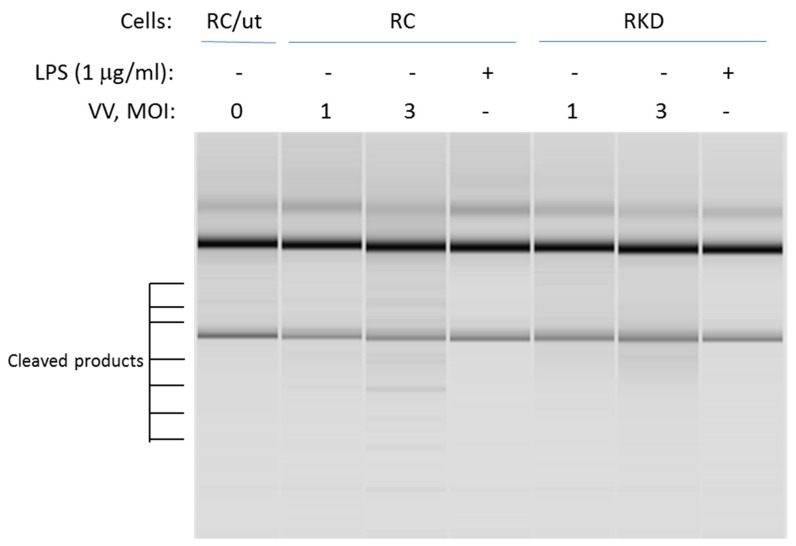
Analysis of RNase L nuclease activity against rRNAs. RNase L wild type and knockdown Raw 264.7 cells were infected by vaccinia virus (VV) with a MOI at 1 and 3; or treated with 1 µg/mL of LPS for 16 h. Total RNA was isolated from the cells using the Trizol reagent according to manufacturer’s instruction. The cleavage of rRNAs in the cells was measured in RNA chips as described in Methods. MOI: multiplicity of infection; RC/ut: Raw Control/Untreated. The experiment was performed twice.

RNase L promotes its biofunction through the 2-5A system. Activated RNase L cleaves both cellular and viral RNAs. It then leads to initiation of the immune response through regulation of the expression of pro- and anti-inflammatory genes. This is accomplished through activating RIG-I-like receptors or NLRP3 by cleaved RNA fragments. To determine if RNase L nuclease activity is necessary for LPS-induced cellular responses, RNase L wild type and knockdown macrophages were infected by vaccinia virus at different multiplicity of infection levels (MOI), or treated with LPS. The cleavage of RNAs was then assessed to determine RNase L activity. As shown in Figure 5, viral infection clearly induced RNA cleavage in wild type macrophages. In comparison, the effect was markedly reduced in RNase L deficient cells. Interestingly, LPS treatment was unable to activate RNase L enzymatic activity in both RNase L wild type and knockdown cells under this condition.

## 4. Discussion

RNase L protein plays an important role in IFN antiviral function, as well as in the control of cell proliferation. RNase L activated by 2-5A directly cleaves viral or cellular RNAs, resulting in inhibition of viral replication and cell growth. In addition, the small RNA fragments thus produced are capable of activating the RIG-I-mediated signaling pathway or NLRP3 inflammasome, leading to innate immune responses [15,16]. RNase L nuclease activity is also necessary for a variety of biological events such as apoptosis, autophagy, and senescence. In this study, we found that RNase L mediated LPS-induced immune responses independent of its nuclease activity. However, regarding the sensitivity of the rRNA cleavage assay, further demonstration of this observation will be continuingly conducted in the lab.

Acute lung injury (ALI)/acute respiratory distress syndrome (ARDS) is a life-threating pathological condition clinically caused by microbial infection or mechanical injury, which triggers an inflammatory response involving different inflammatory cells and pro-inflammatory mediators. Evidence shows that alveolar macrophages (AM) comprise about 95% of airspace leukocytes [21] responsible for the synthesis and release of various inflammatory mediators to promote inflammation in the lung. A variety of cytokines and pro-inflammatory gene products, including TNF-α, IL-1β, IL-6, IL-17, Cox-2, and iNOS, are believed to be involved in the development of ALI. Some immunosuppressive molecules such as IL-10 also participate in the progress of ALI [22]. IL-10 is an anti-inflammatory cytokine, which effectively suppresses immune responses to protect the host. It has been reported that IL-10 may inhibit the TNF-α production in the ALI progress [23]. Previously, we have observed that RNase L is necessary for the expression of IL-10 and other pro- and anti-inflammatory mediators in bone marrow derived macrophages (BMMs) after stimulation with LPS; and plays an important role in BMM functions [20]. Similarly, the levels of IL-10 in tissue extracts and blood from wild type mice were significantly higher than that in RNase L deficient mice after development of ALI induced by LPS. This observation was also demonstrated in cultured macrophages. Studies have shown that treatment of mice with IL-10 is able to reduce mortality about 30% in ALI [23]; and inhibition of IL-10 production exacerbates lung injury caused by LPS [24]. Obviously, more severe ALI progression in RNase L deficient mice may result from reduced expression of IL-10, although the mechanism of RNase L regulation of IL-10 expression remains to be determined.

The immune cell infiltration in our experiments was not significant, although the levels of IL-1β in both plasma and tissue samples were remarkably elevated after mice were exposed to LPS. Cell destruction and tissue damage were obvious. However, the mechanism by which LPS causes the damage in the lung needs to be further investigated. It is well established that pathogen-triggered hypersensitive reactions also cause cell destruction and tissue damage, which require a pre-sensitized (immune) state of the host. In our case, mice were housed in a sterile environment from birth; and cage, food and water were autoclaved. The chance of pre-exposure to pathogens is very low. Thus, other immune reactions may be the major cause of lung tissue damage under LPS stimulation.

LPS activates the TLR4 signaling pathway to induce the expression of pro- and anti-inflammatory genes, leading to immune responses [25,26]. In general, the activation of the downstream targets by TLR4 may be either MyD88-dependent or -independent. The MyD88- dependent pathway activates both the MAPK and IκB/NFκB pathways, while activation of IRF3 is MyD88-independent. Apparently, ablation of RNase L suppressed both MyD88-dependent and -independent pathways in macrophages stimulated by LPS (Figure 4B). Furthermore, the contribution of RNase L to the activation of JNK, ERK, and IκB/NFκB was also observed in MEFs after LPS treatment (Figure 4C–E). Therefore, the involvement of RNase L in the TLR4 signaling pathway is not cell type specific.
